# Predicting programmed death-ligand 1 (PD-L1) expression with fluorine-18 fluorodeoxyglucose ([^18^F]FDG) positron emission tomography/computed tomography (PET/CT) metabolic parameters in resectable non-small cell lung cancer

**DOI:** 10.1007/s00330-024-10651-5

**Published:** 2024-02-22

**Authors:** Daniel Johnathan Hughes, Eleni Josephides, Robert O’Shea, Thubeena Manickavasagar, Carolyn Horst, Sarah Hunter, Philippe Tanière, Daisuke Nonaka, Mieke Van Hemelrijck, James Spicer, Vicky Goh, Andrea Bille, Eleni Karapanagiotou, Gary J. R. Cook

**Affiliations:** 1 Department of Cancer Imaging, School of Biomedical Engineering and Imaging Sciences, King’s College London, 5th Floor Becket House, 1 Lambeth Palace Road, London, SE1 7EU UK; 2 King’s College London & Guy’s and St Thomas’ PET Centre, London, UK; 3 Cancer Centre at Guy’s, Guy’s and St Thomas’ NHS Foundation Trust, London, UK; 4 Department of Radiology, Guy’s and St Thomas’ NHS Foundation Trust, London, UK; 5 Department of Histopathology, University Hospitals Birmingham NHS Foundation Trust, Birmingham, UK; 6 Department of Histopathology, Guy’s and St Thomas’ NHS Foundation Trust, London, UK; 7 School of Cancer and Pharmaceutical Sciences, King’s College London, London, UK

**Keywords:** Positron emission tomography, Carcinoma (Non-Small Cell Lung), Immune checkpoint inhibitors, B7-H1 antigen

## Abstract

**Abstract:**

**Background:**

Programmed death-ligand 1 (PD-L1) expression is a predictive biomarker for immunotherapy in non-small cell lung cancer (NSCLC). PD-L1 and glucose transporter 1 expression are closely associated, and studies demonstrate correlation of PD-L1 with glucose metabolism.

**Aim:**

The aim of this study was to investigate the association of fluorine-18 fluorodeoxyglucose positron emission tomography/computed tomography ([^18^F]FDG-PET/CT) metabolic parameters with PD-L1 expression in primary lung tumour and lymph node metastases in resected NSCLC.

**Methods:**

We conducted a retrospective analysis of 210 patients with node-positive resectable stage IIB–IIIB NSCLC. PD-L1 tumour proportion score (TPS) was determined using the DAKO 22C3 immunohistochemical assay. Semi-automated techniques were used to analyse pre-operative [^18^F]FDG-PET/CT images to determine primary and nodal metabolic parameter scores (including max, mean, peak and peak adjusted for lean body mass standardised uptake values (SUV), metabolic tumour volume (MTV), total lesional glycolysis (TLG) and SUV heterogeneity index (HISUV)).

**Results:**

Patients were predominantly male (57%), median age 70 years with non-squamous NSCLC (68%). A majority had negative primary tumour PD-L1 (TPS < 1%; 53%). Mean SUV_max_, SUV_mean_, SUV_peak_ and SUL_peak_ values were significantly higher (*p* < 0.05) in those with TPS ≥ 1% in primary tumour (*n* = 210) or lymph nodes (*n* = 91). However, ROC analysis demonstrated only moderate separability at the 1% PD-L1 TPS threshold (AUCs 0.58–0.73). There was no association of MTV, TLG and HISUV with PD-L1 TPS.

**Conclusion:**

This study demonstrated the association of SUV-based [^18^F]FDG-PET/CT metabolic parameters with PD-L1 expression in primary tumour or lymph node metastasis in resectable NSCLC, but with poor sensitivity and specificity for predicting PD-L1 positivity ≥ 1%.

**Clinical relevance statement:**

Whilst SUV-based fluorine-18 fluorodeoxyglucose positron emission tomography/computed tomography metabolic parameters may not predict programmed death-ligand 1 positivity ≥ 1% in the primary tumour and lymph nodes of resectable non-small cell lung cancer independently, there is a clear association which warrants further investigation in prospective studies.

**Trial registration:**

Non-applicable

**Key Points:**

• *Programmed death-ligand 1 immunohistochemistry has a predictive role in non-small cell lung cancer immunotherapy; however, it is both heterogenous and dynamic.*

• *SUV-based fluorine-18 fluorodeoxyglucose positron emission tomography/computed tomography ([*^*18*^*F]FDG-PET/CT) metabolic parameters were significantly higher in primary tumour or lymph node metastases with positive programmed death-ligand 1 expression.*

• *These SUV-based parameters could potentially play an additive role along with other multi-modal biomarkers in selecting patients within a predictive nomogram.*

**Supplementary Information:**

The online version contains supplementary material available at 10.1007/s00330-024-10651-5.

## Background

Monoclonal antibodies targeting programmed cell death protein-1 (PD-1) or programmed death-ligand 1 (PD-L1) have revolutionised treatment approaches of several cancers, including non-small cell lung cancer (NSCLC) [1–5]. PD-L1 tumour proportion score (TPS), measured by immunohistochemistry, is a validated biomarker for anti-PD-1/PD-L1 therapies [6, 7]. In advanced NSCLC, a PD-L1 TPS ≥ 50% indicates a likely response to anti-PD-1/PD-L1 therapy, negating the need for cytotoxic combination and its associated toxicities [1]. However, not all PD-L1 expressors respond, temporospatial heterogeneity of expression is well-documented, and there are several PD-L1 assays available; as such, patient selection remains a significant challenge [8, 9].

The neoadjuvant CheckMate 816 trial of nivolumab plus chemotherapy vs chemotherapy alone in early-stage NSCLC demonstrated benefits in pathological complete response rates (24.0% vs 2.2%, respectively) and in median event-free survival (31.6 vs 20.8 months) in stage IB–IIIA NSCLC [10]. The benefit was present across all PD-L1 TPS groups, but event-free survival in those with tumour PD-L1 expression ≥ 1% was improved.

Fluorine-18 fluorodeoxyglucose positron emission tomography/computed tomography ([^18^F]FDG-PET/CT) is an important imaging modality used to stage NSCLC [11]. [^18^F]FDG accumulation correlates with the expression of glucose transporter 1 (GLUT1), hexokinase II (HK2) and hypoxia-inducible factor 1-alpha (HIF-1α), with roles in glucose metabolism and hypoxia, respectively [12]. Several studies have demonstrated the correlation of [^18^F]FDG uptake, as well as GLUT1, HK2 and HIF-1α expression, with PD-L1 expression [13–15]. It is therefore unsurprising that [^18^F]FDG-PET/CT metabolic parameters have been associated with PD-L1 immunohistochemistry and/or response to anti-PD-1/PD-L1 therapy [16–24].

The majority of reported studies have been conducted in advanced disease and/or focus on PD-L1 expression of the primary lesion only. Our aim, therefore, was to investigate the role of various metabolic parameters, including those of tumour burden, measured using pre-operative [^18^F]FDG-PET/CT, in predicting PD-L1 expression of primary tumour and nodal metastases in patients with resectable NSCLC.

## Methods

**Fig. 1:**
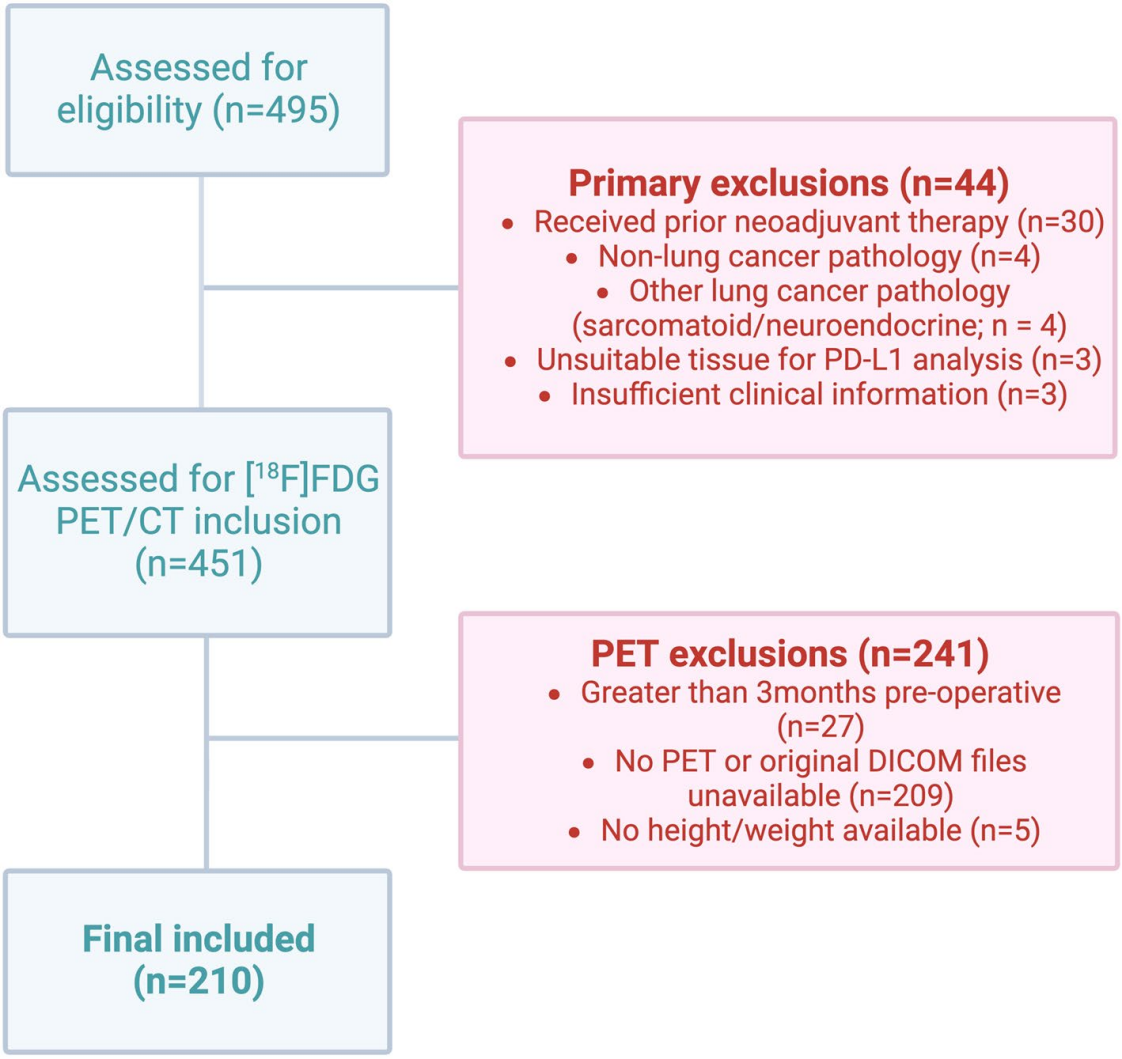
Flow (CONSORT) diagram representing study inclusion criteria. *Created with BioRender.com*

The study was approved by UK Research Ethics Committee (UK IRAS 228790) and within the Guy’s Cancer Cohort (ref: 18/NW/0297) [25]. We conducted a retrospective analysis of 495 consecutive patients with node-positive (stage IIB–IIIB, IASLC TNM 8th ed.) and treatment-naïve NSCLC who were referred to and underwent primary resection at a tertiary centre between February 2009 and October 2018 (Fig. 1). Eligibility criteria for the study included age ≥ 18 years, histological diagnosis of NSCLC and pathologically confirmed N1/N2 lymph node involvement. Patients had all been treated with curative-intent surgical resection of the primary lung tumour, and hilar and/or mediastinal lymphadenectomy. Patients were excluded if there was no available or assessable pre-operative [^18^F]FDG PET/CT imaging within 3 months, clinical data and/or histopathological specimens are not available or they received pre-operative systemic anti-cancer and/or radio-therapy. Electronic medical records were reviewed for demographics including age, smoking history, diagnosis and surgery dates, histology and pathological stage.

### Histopathological assessment

Resected specimen haematoxylin and eosin slides were assessed to confirm the histopathological diagnosis and quality/quantity of tumour to select representative formalin-fixed paraffin-embedded tissue blocks from the primary tumour and nodal metastases where present. If more than one lymph node was involved, the node furthest from the primary tumour was used.

PD-L1 immunohistochemistry was performed using the 22C3 pharmDx assay on the Dako Autostainer Link 48 (Agilent Technologies Inc.). A minimum of 100 viable tumour cells were required for analysis; stained slides were assessed by two independent consultant histopathologists [26]. The TPS was calculated as the percentage of PD-L1-positive tumour cells relative to all viable tumour cells in the specimen. Patients were subsequently categorised as TPS < 1%, 1–49% or ≥ 50%, with < or ≥ 1% and < or ≥ 50% thresholds being commonly used for treatment decisions in early-stage and late-stage disease settings [1–7, 10, 27, 28].

### *[*^*18*^*F]FDG PET/CT protocol*

[^18^F]FDG PET/CT scans were performed at the local referring centre or at King’s College London & Guy’s and St Thomas’ PET Centre (London, UK) according to local protocols and in accordance with current guidelines [29]. Patients were fasted at least 6 h and glucose levels confirmed < 180 mg/dL prior to imaging. Patients were injected with 2.5–3.5 MBq/kg FDG (median activity 336 MBq [range 194–436]) with images acquired at 60 min post-injection. PET images were reconstructed using iterative techniques and CT for attenuation correction.

### Image analysis

**Fig. 2:**
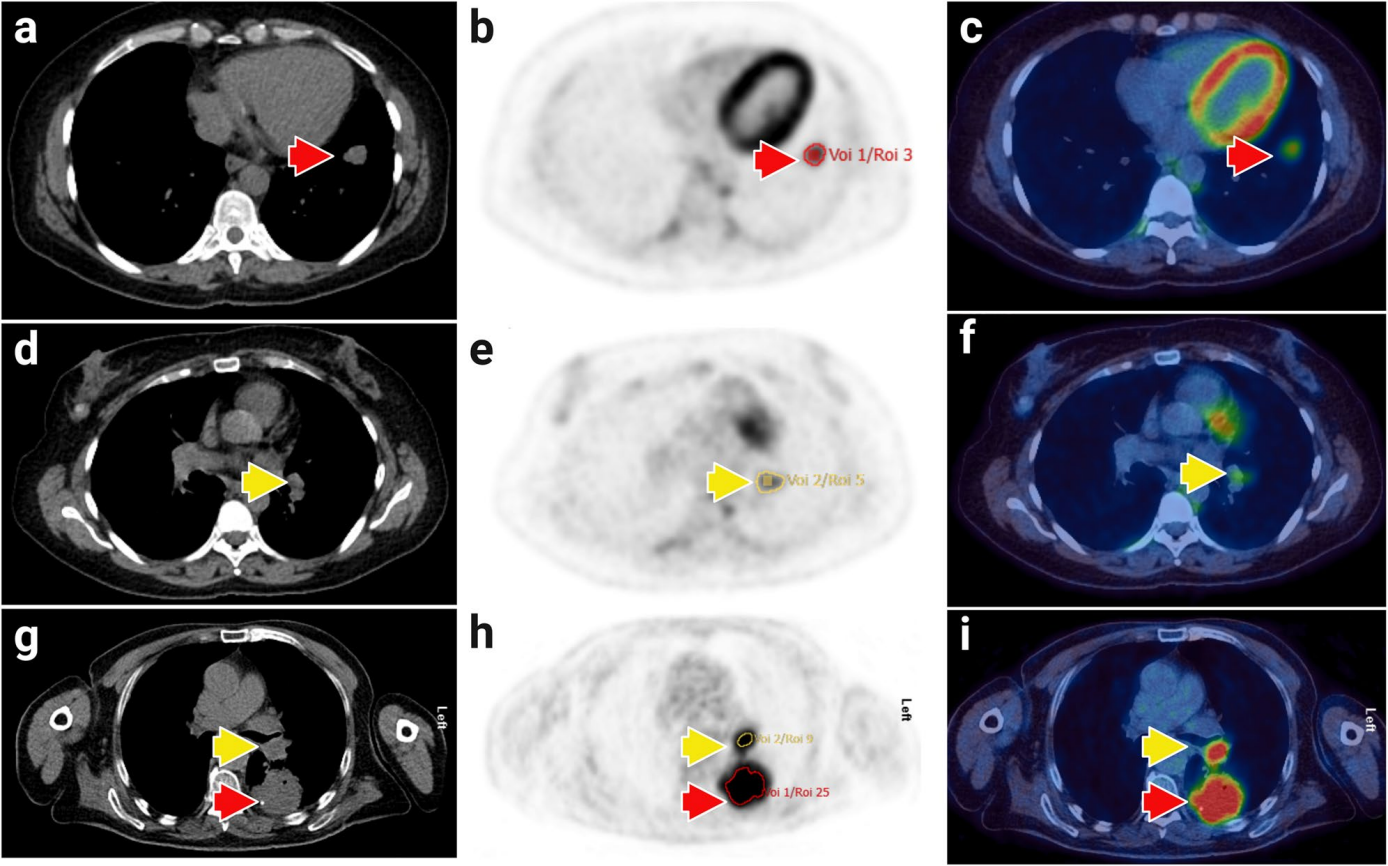
Image analysis example. **a**–**f** Patient 1 with T1bN2M0 (stage IIIA) non-squamous NSCLC with negative PD-L1 TPS < 1% in both primary and nodal disease. **a** CT image of primary tumour for anatomical correlation; **b** PET image of primary tumour depicting VOI with an SUV_max_ 6.75, SUV_mean_ 4.03, SUV_peak_ 4.84, SUL_peak_ 3.16, MTV 2.79 cm^3^, TLG 11.24 and HISUV 1.67; and (**c**) fused axial PET/CT images showing primary tumour. **d** CT image of lymph node for anatomical correlation; **e** PET image of lymph node metastasis depicting VOI with SUV_max_ 5.19, SUV_mean_ 2.91, SUV_peak_ 4.05, SUL_peak_ 2.65, MTV 7.65 cm^3^, TLG 22.29 and HISUV 1.78; **f** fused axial PET/CT images. **g**–**i** Patient 2 with T3N2M0 (stage IIIB) non-squamous NSCLC with positive (high, ≥ 50%) PD-L1 TPS 90% in both primary and nodal metastasis. **g** CT image of primary tumour and lymph node metastasis for anatomical correlation; **h** PET image of primary tumour (red arrow) VOI with SUV_max_ 24.55, SUV_mean_ 12.49, SUV_peak_ 20.31, SUL_peak_ 19.12, MTV 80.22 cm^3^, TLG 1002.11 and HISUV 1.97, and lymph node metastasis (yellow arrow) VOI with SUV_max_ 16.59, SUV_mean_ 12.71, SUV_peak_ 15.45, SUL_peak_ 13.08, MTV 6.07 cm^3^, TLG 77.19 and HISUV 1.31; and (**i**) fused axial PET/CT images. Red arrows denote primary tumour; yellow arrows denote lymph node metastasis. *Created with BioRender.com*

Images were reviewed by an experienced nuclear medicine researcher using Hermes GOLD™ (Hermes Medical Solutions). Volumes of interest (VOI), including the primary lung tumour and associated thoracic lymph nodes, were identified with CT correlation (Fig. 2). Malignant lesions (primary and lymph nodes) analysed on imaging were matched with their respective histopathologically assessed lesions, to allow correlation of histological PD-L1 status with metabolic parameters. A semi-automated segmentation method was used to delineate the VOI, with the metabolic tumour volume (MTV, cm^3^) established using a 40% threshold of the maximum standardised uptake value (SUV_max_). The mean SUV (SUV_mean_), peak SUV (SUV_peak_) and SUV_peak_ adjusted for lean body mass (SUL_peak_) were also recorded. In addition, primary tumour and thoracic lymph node total lesion glycolysis (TLG = MTV × SUV_mean_) and SUV-based heterogeneity index (HISUV = SUV_max_ ÷ SUV_mean_) were calculated.

### Statistical analysis

Continuous variables are presented as means with the standard error; categorical variables are presented with absolute and relative frequencies. Categorical data were analysed using the chi-squared test. Normality of continuous variables was assessed using the Shapiro-Wilk test. Correlations between PD-L1 TPS and metabolic parameters were assessed with Spearman’s rank correlation coefficient. Parametric data, according to PD-L1 TPS groups, were analysed using one-way ANOVA, whilst non-parametric data were analysed using the Mann-Whitney *U* test to compare two groups, or Kruskal-Wallis with Dunn’s multiple comparison test for three or more groups. *p* values are two-sided with significance as *α *= 0.05. Youden’s (*J*) statistic was used to determine the optimal cut-off point from receiver operating characteristic (ROC) curves with equal weight given to sensitivity and specificity. Multiple logistic regression was performed for PD-L1 expression controlling for age, sex, smoking history, histology, tumour (T), nodal (N) stage and SUV-based metabolic parameters of interest. Data were analysed and individual graphs created with GraphPad Prism v9.5.1 for macOS (GraphPad Software). Figures were generated using BioRender.com.

## Results

Of 495 consecutive patients, 210 cases met the inclusion criteria; the most common reason for exclusion being PET and/or original DICOM files not available (*n* = 209) (Fig. 1). Patients’ demographic and clinical characteristics are demonstrated in Table 1, and Supplementary Tables 1–5. Included patients were predominantly male (*n* = 120, 57%), median age 70 years (range 40–89 years). A majority had pathologically confirmed non-squamous NSCLC (*n* = 143, 68%). Most patients had either pathological stage IIB (*n* = 81, 39%) or IIIA (*n* = 96, 46%) disease. The median days pre-operatively for [^18^F]FDG PET/CT being performed were 49 days (range 1–93 days).

**Table 1:** Clinical characteristics of all included patients with non-small cell lung cancer by primary tumour PD-L1 tumour proportion score (TPS). *p* values represent between TPS group comparisons

Clinical characteristic	All	Primary tumourPD-L1 < 1%	Primary tumourPD-L1 1–49%	Primary tumourPD-L1 ≥ 50%	*p* value
Number (% of total)	**210 (100)**	111 (53)	74 (35)	25 (12)	
Age at surgery					0.83
Mean (years)	**69**	69	69	70	
Median (years)	**70**	70	69	72	
Range (years)	**40–89**	45–89	40–86	46–86	
Sex, *n* (%)					0.83
Female	**90 (43)**	46 (41)	32 (43)	12 (48)	
Male	**120 (57)**	65 (59)	42 (57)	13 (52)	
Smoking history, *n* (%)					0.73
Current smoker	**186 (89)**	98 (88)	65 (88)	23 (92)	
Ex smoker	**8 (4)**	4 (4)	3 (4)	1 (4)	
Never smoker	**10 (5)**	8 (7)	2 (3)	0 (0)	
Unknown	**6 (3)**	1 (1)	4 (5)	1 (4)	
Histopathology, *n* (%)					0.47
Non-squamous*	**143 (68)**	77 (69)	47 (64)	19 (76)	
Squamous cell carcinoma	**67 (32)**	34 (31)	27 (36)	6 (24)	
Lymph node PD-L1 score, *n* (%)					**< 0.001**
< 1	**133 (63)**	103 (93)	25 (34)	5 (20)	
1–49	**60 (29)**	7 (6)	44 (59)	9 (36)	
≥ 50	**17 (8)**	1 (1)	5 (7)	11 (44)	
Primary tumour location, *n* (%)					0.79
Left	**88 (42)**	45 (41)	31 (42)	12 (48)	
Right	**122 (58)**	66 (59)	43 (58)	13 (52)	
Lymphovascular invasion, *n* (%)					0.44
Yes	**108 (51)**	55 (50)	37 (50)	16 (64)	
No	**100 (48)**	54 (49)	37 (50)	9 (36)	
Unknown	**2 (1)**	2 (2)	0 (0)	0 (0)	
Primary tumour max diameter (mm)					0.59
Median	**40**	38	42	35	
Range	**5–150**	11–150	5–98	10–90	
Pathological T stage, *n* (%)					0.48
1 (a–c)	**38 (18)**	22 (20)	12 (16)	4 (16)	
2 (a, b)	**101 (48)**	49 (44)	35 (48)	17 (68)	
3	**50 (24)**	28 (25)	19 (26)	3 (12)	
4	**21 (10)**	12 (11)	8 (11)	1 (4)	
Pathological N stage, *n* (%)					0.23
1	**118 (56)**	61 (55)	39 (53)	18 (72)	
2	**92 (44)**	50 (45)	35 (47)	7 (28)	
Pathological M stage, *n* (%)					0.83
M0	**208 (99)**	110 (99)	73 (99)	25 (100)	
M1a	**2 (1)**	1 (1)	1 (1)	0 (0)	
Pathological stage, *n* (%)					0.20
IIB	**81 (39)**	40 (36)	25 (34)	16 (64)	
IIIA	**96 (46)**	52 (47)	36 (49)	8 (32)	
IIIB	**31 (15)**	18 (16)	12 (16)	1 (4)	
IVA	**2 (1)**	1 (1)	1 (1)	0 (0)	

*Non-squamous includes adenocarcinoma, adenosquamous carcinoma, large cell carcinoma and NSCLC—not otherwise specified

A majority of patients had negative (TPS < 1%) PD-L1 expression determined by immunohistochemistry in the primary tumour (*n* = 111, 53%). Seventy-four (35%) had low (TPS 1–49%) PD-L1 expression and 25 (12%) had high (TPS ≥ 50%) PD-L1 expression. There was no statistical difference in clinical characteristics including age, sex, histopathological, tumour location or stage between the groups (Table 1). The only significant difference between the groups was the nodal metastasis PD-L1 TPS (*p* < 0.001). This was also the case when comparing clinical characteristics by the nodal metastasis PD-L1 TPS group, with the only significant difference being primary tumour PD-L1 TPS (*p* < 0.001; Supplementary Table 1). This was related to the inter-lesional heterogeneity between primary tumour and nodal metastases within individuals. The frequency of heterogeneity based on primary tumour PD-L1 TPS groups of < 1%, 1–49% and ≥ 50% was 7% (*n* = 8 of 111), 41% (*n* = 30 of 74) and 56% (*n* = 14 of 25), respectively.

### Metabolic parameters and PD-L1 expression by TPS

Correlations between TPS and measured metabolic parameters of primary tumour or nodal metastasis are summarised in Table 2. There was a positive correlation between TPS and primary tumour SUV_max_ (Spearman’s rho (*r*) = 0.20; *p* < 0.05), SUV_mean_ (*r* = 0.20; *p* < 0.05), SUV_peak_ (*r* = 0.16;* p* < 0.05) and SUL_peak_ (*r* = 0.15; *p* < 0.05). Similarly, there was a positive correlation between TPS and nodal metastasis SUV_max_ (*r* = 0.30; *p* < 0.05) and SUV_mean_ (*r* = 0.35; *p* < 0.05). However, there was no correlation for SUV_peak_ nor SUL_peak_. There was no correlation between TPS and metabolic parameters of tumour burden and heterogeneity (MTV, TLG, HISUV) of the primary tumour nor nodal metastasis.

**Table 2:** Correlation of PD-L1 TPS with [^18^F]FDG metabolic parameter scores for primary lung tumour or involved lymph nodes. The *n* varies between groups and individual parameters, for example, peak is only measurable in lesions with a minimum 1 cm^3^ volume

PET metabolic parameter	Spearman’s rho (*r*)	95% confidence intervals	*p* value
**SUV**_**max**_			
Primary tumour (*n* = 210)	0.20	0.07–0.33	**0.003**
Lymph node (*n* = 91)	0.30	0.10–0.49	**0.003**
**SUV**_**mean**_			
Primary tumour (*n* = 207)	0.20	0.07–0.33	**0.003**
Lymph node (*n* = 89)	0.35	0.15–0.53	**< 0.001**
**SUV**_**peak**_			
Primary tumour (*n* = 188)	0.16	0.01–0.30	**0.03**
Lymph node (*n* = 45)	0.26	− 0.05 to 0.52	0.09
**SUL**_**peak**_			
Primary tumour (*n* = 185)	0.15	0.00–0.29	**0.04**
Lymph node (*n* = 43)	0.22	− 0.09 to 0.50	0.15
**MTV**			
Primary tumour (*n* = 207)	0.03	− 0.11 to 0.17	0.69
Lymph node (*n* = 89)	0.06	− 0.15 to 0.27	0.55
**TLG**			
Primary tumour (*n* = 207)	0.07	− 0.07 to 0.20	0.34
Lymph node (*n* = 89)	0.14	− 0.08 to 0.34	0.19
**HISUV**			
Primary tumour (*n* = 207)	− 0.09	− 0.23 to 0.05	0.18
Lymph node (*n* = 89)	0.03	− 0.19 to 0.24	0.78

### Metabolic parameters and PD-L1 expression by TPS categories

**Fig. 3:**
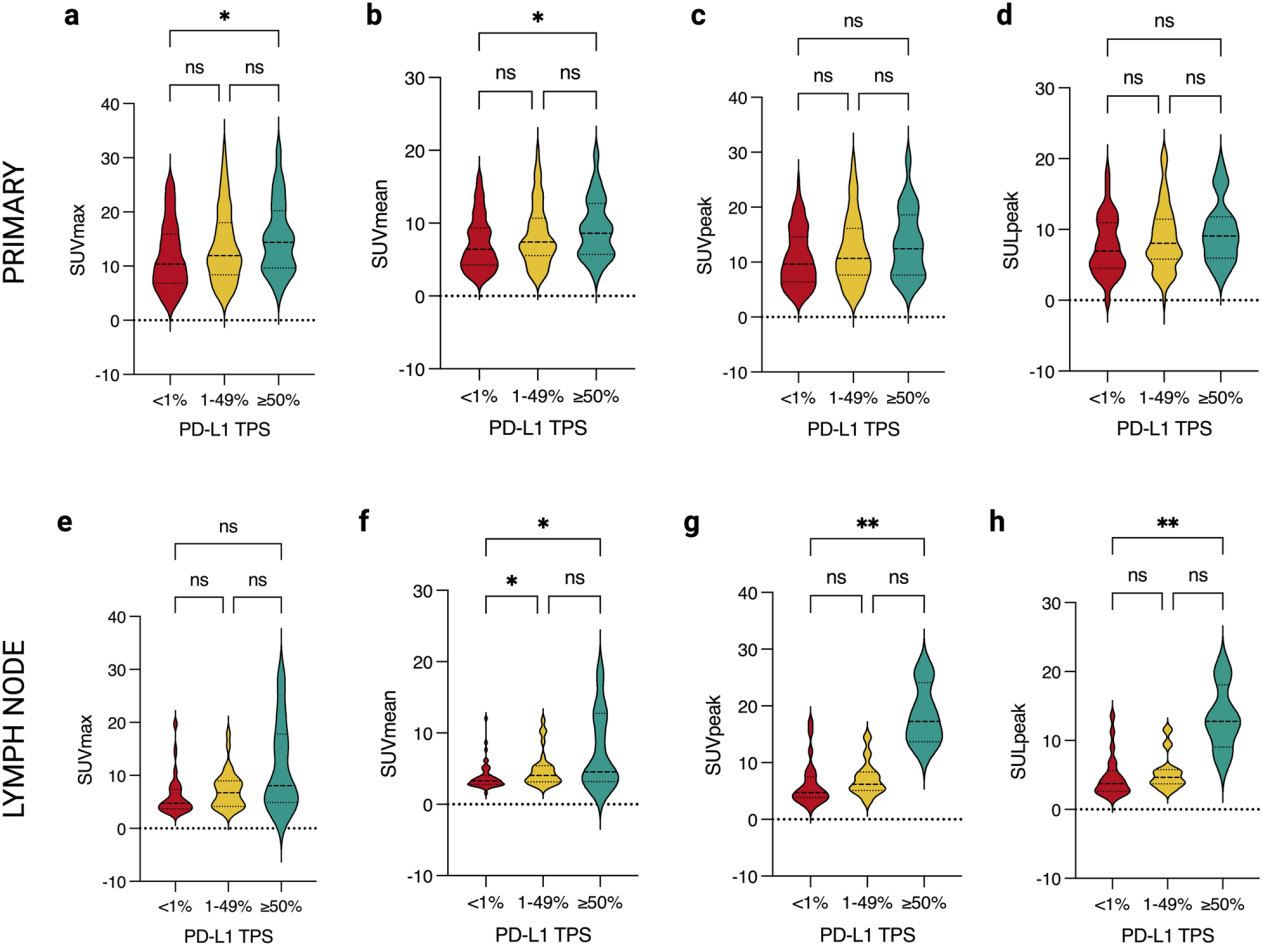
Violin plots displaying primary lung tumour (**a**–**d**) and malignant lymph node (**e**–**h**) SUV-based metabolic parameter median and lower/upper quartiles (dashed lines) of PD-L1 TPS groups of < 1%, 1–49% and ≥ 50%. Metabolic parameters of (**a**) primary SUV_max_ (*p* < 0.05, *H* test = 7.60), (**b**) primary SUV_mean_ (*p* < 0.05, *H* test = 7.72), (**c**) primary SUV_peak_ (*p* = 0.13, *H* test = 4.10), (**d**) primary SUL_peak_ (*p* = 0.15, *H* test = 3.85), (**e**) lymph node SUV_max_ (*p* < 0.05, *H* test = 7.24), (**f**) lymph node SUV_mean_ (*p* < 0.05, *H* test = 9.85), (**g**) lymph node SUV_peak_ (*p* < 0.05, *H* test = 11.17) and (**h**) lymph node SUL_peak_ (*p* < 0.05, *H* test = 9.74). Lines above the plots demonstrate Dunn’s multiple comparison tests between two individual TPS groups, where ns is non-significant (*p* > 0.05) and * represents the level of significance (* is *p* < 0.05; ** is *p* < 0.01 and *** is *p* < 0.001). *Individual graphs created in GraphPad Prism, and figure created with BioRender.com*

The mean metabolic parameter scores with their standard error for both primary tumour and nodal metastases according to all TPS categories are summarised in Table 3. The mean SUV_max_ of primary tumour (*n* = 210) increased according to the < 1%, 1–49% and ≥ 50% TPS groups, at 11.75, 13.66 and 15.19, respectively (*p* = 0.02). On multiple comparison testing, significance held between TPS groups < 1% and ≥ 50%, but not between 1–49% group with either the < 1% or ≥ 50% group (Fig. 3). There was a similar trend but with lower mean scores for involved lymph nodes (*n* = 91) with mean SUV_max_ of 6.21, 7.26 and 11.50 for lymph node TPS groups of < 1%, 1–49% and ≥ 50%, respectively (*p* = 0.03), but with no significant difference between individual TPS groups (Fig. 3).

**Table 3:** Mean metabolic parameter scores and their standard error (SE), for primary lung tumour or involved lymph nodes, by their immunohistochemistry PD-L1 TPS groups. The *n* varies between groups and individual parameters, for example, peak is only measurable in lesions with a minimum 1 cm^3^ volume. Kruskal-Wallis *p* values presented for each parameter in primary lung tumour or lymph node metastasis (significant in bold)

PET metabolic parameter	PD-L1 TPS < 1%	PD-L1 TPS 1–49%	PD-L1 TPS ≥ 50%	*Kruskal-Wallis p value*
**SUV**_**max**_				
Primary tumour, *n*	111	74	25	
*Mean (SE)*	11.75 (0.58)	13.66 (0.78)	15.19 (1.32)	**0.02**
Lymph node, *n*	50	31	10	
*Mean (SE)*	6.21 (0.55)	7.26 (0.63)	11.50 (2.64)	**0.03**
**SUV**_**mean**_				
Primary tumour, *n*	109	73	25	
*Mean (SE)*	7.16 (0.35)	8.37 (0.49)	9.23 (0.82)	**0.02**
Lymph node, *n*	49	30	10	
*Mean (SE)*	3.82 (0.26)	4.84 (0.44)	7.48 (1.71)	**0.007**
**SUV**_**peak**_				
Primary tumour, *n*	95	70	23	
*Mean (SE)*	10.76 (0.54)	12.21 (0.74)	13.44 (1.30)	0.13
Lymph node, *n*	23	13	4	
*Mean (SE)*	6.21 (0.82)	7.16 (0.85)	18.36 (2.76)	**0.004**
**SUL**_**peak**_				
Primary tumour, *n*	95	67	23	
*Mean (SE)*	7.91 (0.41)	8.89 (0.55)	9.66 (0.90)	0.15
Lymph node, *n*	22	12	4	
*Mean (SE)*	4.73 (0.65)	5.35 (0.76)	13.31 (2.45)	**0.008**
**MTV**				
Primary tumour, *n*	109	73	25	
*Mean (SE)*	26.99 (4.19)	22.15 (2.95)	20.74 (5.28)	0.80
Lymph node, *n*	49	30	10	
*Mean (SE)*	3.32 (0.50)	3.33 (0.57)	11.79 (7.19)	0.71
**TLG**				
Primary tumour, *n*	109	73	25	
*Mean (SE)*	219.9 (34.29)	213.1 (35.17)	221.0 (59.28)	0.82
Lymph node,* n*	49	30	10	
*Mean (SE)*	15.17 (3.10)	17.17 (4.14)	117.4 (66.42)	0.47
**HISUV**				
Primary tumour, *n*	109	73	25	
*Mean (SE)*	1.66 (0.01)	1.66 (0.01)	1.66 (0.02)	0.30
Lymph node, *n*	49	30	10	
*Mean (SE)*	1.54 (0.05)	1.55 (0.05)	1.53 (0.08)	0.97

The SUV_mean_ of the primary tumour also increased according to TPS groups, with a mean SUV_mean_ of 7.16, 8.37 and 9.23, respectively (*p* = 0.02). Multiple comparison testing demonstrated significant difference (*p* < 0.05) between the < 1% and ≥ 50% primary tumour TPS groups only. A similar significant trend was seen with mean SUV_mean_ values of 3.82, 4.84 and 7.48 for nodal metastases according to TPS groups < 1%, 1–49% and ≥ 50%, respectively (*p* = 0.007). There was no significant difference in SUV_mean_ scores between nodal TPS groups of 1–49% and ≥ 50%.

Although there was a trend toward increased mean SUV_peak_ of the primary lung tumour with increasing TPS group, this was not significant (*p* = 0.13). However, the trend of increased mean SUV_peak_ of the nodal metastasis with increasing lymph node TPS category was significant (*p* = 0.004). When adjusting SUV_peak_ for lean body mass, the mean SUL_peak_ values for the primary lung tumour did not significantly differ between TPS groups (*p* = 0.15). The trend of increased mean SUL_peak_ with increasing TPS group in nodal metastases was significant (*p* = 0.008).

There were no statistically significant differences between all TPS groups of the primary lung tumour and nodal metastases for mean MTV (*p* = 0.80; *p* = 0.71, respectively), TLG (*p* = 0.82; *p* = 0.47) and HISUV (*p* = 0.30; *p* = 0.97) (Supplementary Figure 1).

### Metabolic parameters and PD-L1 expression by PD-L1 TPS of < or ≥ 1%

The clinical characteristics of patients using either primary lung tumour or nodal metastases by TPS groups < or ≥ 1% are presented in Supplementary Tables 2 and 3. The only characteristic of significance between TPS groups is the nodal metastasis and primary tumour TPS groups, respectively. The mean metabolic parameter scores with their standard error for both primary tumour and nodal metastases according to PD-L1 TPS categories of < or ≥ 1% are summarised in Table 4.

**Table 4:** Mean metabolic parameter scores and their standard error (SE), for primary lung tumour or involved lymph nodes, by PD-L1 TPS above or below the 1% threshold for positive expression. The *n* varies between groups and individual parameters, for example, peak is only measurable in lesions with a minimum 1 cm^3^ volume. Mann-Whitney *p* values presented for each parameter in primary lung tumour or lymph node metastasis (significant in bold)

PET metabolic parameter	PD-L1 TPS < 1%	PD-L1 TPS ≥ 1%	*Mann-Whitney p value*
**SUV**_**max**_			
Primary tumour, *n*	111	99	
*Mean (SE)*	11.75 (0.58)	14.05 (0.67)	**0.01**
Lymph node, *n*	50	41	
*Mean (SE)*	6.21 (0.55)	8.29 (0.83)	**0.01**
**SUV**_**mean**_			
Primary tumour, *n*	109	98	
*Mean (SE)*	7.16 (0.35)	8.59 (0.42)	**0.009**
Lymph node, *n*	49	40	
*Mean (SE)*	3.82 (0.26)	5.50 (0.56)	**0.002**
**SUV**_**peak**_			
Primary tumour, *n*	98	94	
*Mean (SE)*	10.43 (0.55)	12.38 (0.65)	**0.04**
Lymph node, *n*	23	17	
*Mean (SE)*	6.21 (0.82)	9.80 (1.47)	**0.01**
**SUL**_**peak**_			
Primary tumour, *n*	98	91	
*Mean (SE)*	7.67 (0.42)	8.98 (0.48)	**0.05**
Lymph node, *n*	23	16	
*Mean (SE)*	4.53 (0.66)	7.34 (1.19)	**0.02**
**MTV**			
Primary tumour, *n*	109	98	
*Mean (SE)*	26.99 (4.09)	21.79 (2.57)	0.88
Lymph node, *n*	49	40	
*Mean (SE)*	3.32 (0.50)	5.44 (1.87)	0.81
**TLG**			
Primary tumour, *n*	109	98	
*Mean (SE)*	219.90 (34.29)	215.10 (30.10)	0.54
Lymph node,* n*	49	40	
*Mean (SE)*	15.17 (3.10)	42.23 (17.67)	0.34
**HISUV**			
Primary tumour, *n*	109	98	
*Mean (SE)*	1.66 (0.01)	1.66 (0.01)	0.12
Lymph node, *n*	49	40	
*Mean (SE)*	1.54 (0.05)	1.55 (0.04)	0.83

**Fig. 4:**
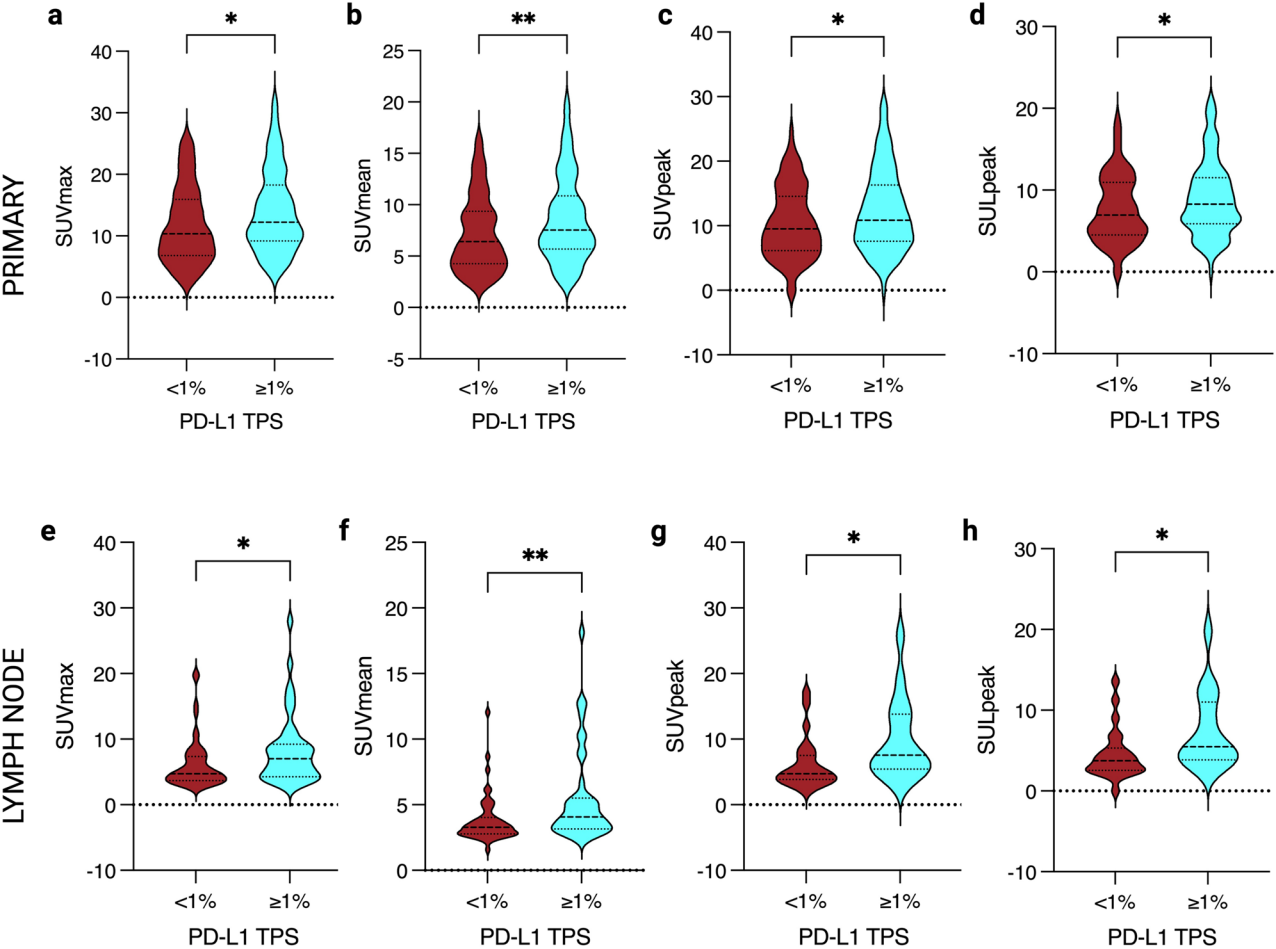
Violin plots displaying primary lung tumour (**a**–**d**) and malignant lymph node (**e**–**h**) metabolic parameter median and lower/upper quartiles (dashed lines) of PD-L1 TPS groups of < 1% and ≥ 1%. Metabolic parameters of (**a**) primary SUV_max_ (*p* < 0.05), (**b**) primary SUV_mean_ (*p* < 0.01), (**c**) primary SUV_peak_ (*p* < 0.05), (**d**) primary SUL_peak_ (*p* = 0.05), (**e**) lymph node SUV_max_ (*p* < 0.05), (**f**) lymph node SUV_mean_ (*p* < 0.05), (**g**) lymph node SUV_peak_ (*p* < 0.05) and (**h**) lymph node SUL_peak_ (*p* < 0.05). Lines above the plots demonstrate the Mann-Whitney *U* test *p* values between the two TPS groups, where ns is non-significant (*p* > 0.05) and * represents the level of significance (* is *p* < 0.05; ** is *p* < 0.01 and *** is *p* < 0.001). *Individual graphs created in GraphPad Prism, and figure created with BioRender.com*

The SUV_max_ of primary tumour (*n* = 210) increased according to the TPS group < or ≥ 1%, with mean SUV_max_ of 11.75 and 14.05, respectively (*p* = 0.01; Fig. 4). Mean SUV_max_ measurements were also significantly higher in nodal metastases with PD-L1 ≥ 1% (8.29) compared to those with no PD-L1 expression < 1% (6.21) (*p* = 0.01). The SUV_mean_ of the primary tumour was also higher in those with a TPS ≥ 1% at 8.59 compared to those with TPS < 1% at 7.16 (*p* = 0.009). Similarly, the mean SUV_mean_ measurements were higher in the TPS ≥ 1% nodal metastasis group at 5.50 compared to the TPS < 1% group at 3.82 (*p* = 0.002).

The mean SUV_peak_ of primary lung tumour with a TPS ≥ 1% was higher at 12.38 compared to those with a TPS < 1% at 10.43 (*p* = 0.04). A statistical difference of SUV_peak_ measurements was also present for nodal metastases in those with TPS ≥ 1% at 9.80 compared to those with TPS < 1% at 6.21 (*p* = 0.01). Adjusted for lean body mass, mean SUL_peak_ measurements were also higher in primary tumours with positive TPS ≥ 1% at 8.98 compared to those with TPS < 1% at 7.67 (*p* = 0.05). Similarly, there was a significant difference in mean SUL_peak_ measurements for nodal metastases with TPS ≥ 1% at 7.34 compared to those with TPS < 1% at 4.53 (*p* = 0.02).

There was no statistically significant difference between primary tumour TPS < 1% and ≥ 1% for the mean MTV (*p* = 0.88), TLG (*p* = 0.54) and HISUV (*p* = 0.12). There was also no difference between the nodal metastasis TPS groups < or ≥ 1% for mean MTV (*p* = 0.81), TLG (*p* = 0.34) and HISUV (*p* = 0.83) (Table 4; Supplementary Figure 2).

**Fig. 5:**
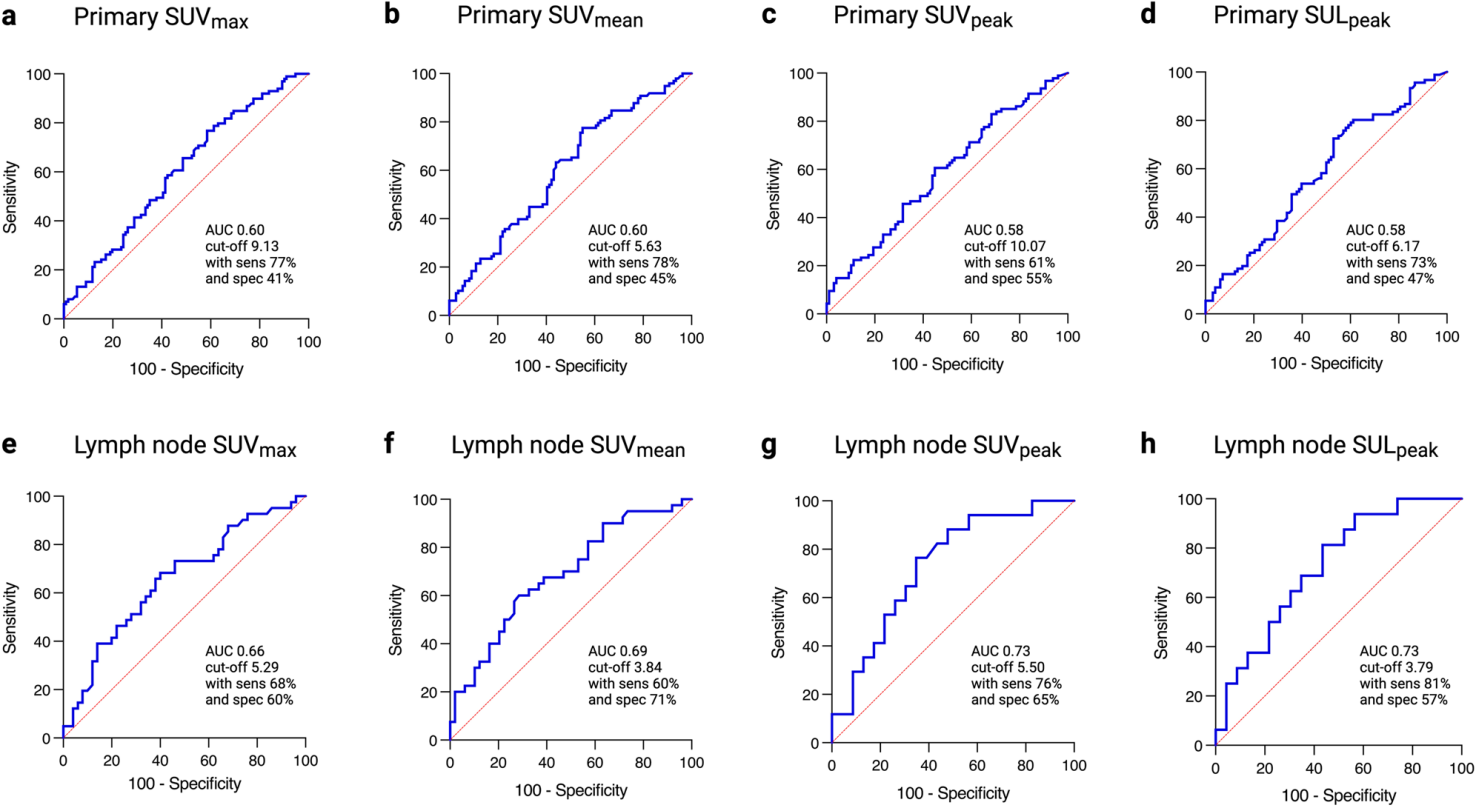
ROC curves displaying both primary tumour (**a**–**d**) and malignant lymph node (**e**–**h**) significant metabolic parameters for PD-L1 TPS of < or ≥ 1%. Primary tumour (**a**) SUV_max_ (AUC 0.60; 95% CI 0.53–0.68; *p* = 0.01; cut-off value 9.13 with sensitivity 77% and specificity 41%), (**b**) SUV_mean_ (AUC 0.60; 95% CI 0.53–0.68; *p* = 0.009; cut-off value 5.63 with sensitivity 78% and specificity 45%), (**c**) SUV_peak_ (AUC 0.58; 95% CI 0.50–0.66; *p* = 0.04; cut-off value 10.07 with sensitivity 61% and specificity 55%) and (**d**) SUL_peak_ (AUC 0.58; 95% CI 0.50–0.66; *p* = 0.05; cut-off value 6.17 with sensitivity 73% and specificity 47%). Lymph node metastasis (**e**) SUV_max_ (AUC 0.66; 95% CI 0.54–0.77; *p* = 0.01; cut-off value 5.29 with sensitivity 68% and specificity 60%), (**f**) SUV_mean_ (AUC 0.69; 95% CI 0.58–0.80; *p* = 0.003; cut-off value 3.84 with sensitivity 60% and specificity 71%), (**g**) SUV_peak_ (AUC 0.73; 95% CI 0.58–0.89; *p* = 0.01; cut-off value 5.50 with sensitivity 76% and specificity 65%) and (**h**) SUL_peak_ (AUC 0.73; 95% CI 0.57–0.88; *p* = 0.02; cut-off value 3.79 with sensitivity 81% and specificity 57%). *Individual graphs created in GraphPad Prism, and figure created with BioRender.com*

To determine the sensitivity and specificity of the metabolic parameters associated with TPS immunohistochemistry positivity at the 1% threshold, ROC and area under the curve (AUC) analyses were performed (Fig. 5). The AUCs for metabolic parameters SUV_max_, SUV_mean_, SUV_peak_ and SUL_peak_ for primary tumour were in range 0.58–0.60, and for nodal metastases in range 0.66–0.73. The AUC for primary tumour SUV_max_ at the 1% positive threshold was 0.60, with SUV_max_ > 9.13 providing a sensitivity of 77% and specificity of 41%. Similarly, the AUC for nodal SUV_max_ at the 1% threshold was 0.66, with SUV_max_ > 5.29 providing a sensitivity of 68% and specificity of 60%. Multivariate analysis was also performed for PD-L1 expression at the 1% threshold, with no factors of interest, including SUV_max_ and SUV_mean_, determined as independent predictors for the primary tumour. However, both age and SUV_mean_ were demonstrated as significant independent predictors for malignant lymph node PD-L1 expression < or ≥ 1% (Supplementary Table 6).

### Metabolic parameters and PD-L1 expression by PD-L1 TPS of < or ≥ 50%

The clinical characteristics of patients using either primary lung tumour or nodal metastases by TPS groups less than or greater than/equal to 50% are presented in Supplementary Tables 4 and 5. The mean metabolic parameter scores with their standard error for both primary tumour and lymph node metastases according to TPS categories of < or ≥ 50% are summarised in Supplementary Table 7. The results were more varied according to the 50% positivity threshold; e.g. the mean SUV_max_ was statistically higher in those with primary tumour TPS of ≥ 50% but not in nodal metastases (Supplementary Figures 3 and 4).

## Discussion

This is the first study, to our knowledge, that demonstrates the association of semi-quantitative [^18^F]FDG-PET/CT metabolic parameters, namely SUV_max_, SUV_mean_, SUV_peak_ and SUL_peak_, with PD-L1 expression in *both* primary tumour and nodal metastases in resectable NSCLC. Mean values of SUV-based metabolic parameters, including SUV_max_, SUV_mean_, SUV_peak_ and SUL_peak_, all increased by TPS groups, but these differences were not significant on correcting for multiple comparisons. Using a PD-L1 positivity threshold of 1%, which is standard for the 22C3 assay used, all of these metabolic parameters were significantly higher in the TPS ≥ 1% group. Whilst it is possible that these parameters could be used to predict TPS groups as measured by immunohistochemistry, the sensitivity and specificity of these measurements were poor, for both primary tumour and nodal metastases. Using primary tumour SUV_mean_ > 5.63 as a cut-off for TPS positivity ≥ 1%, the sensitivity was moderate at 78% but specificity was low at 45%, reflecting multifactorial causes for [^18^F]FDG uptake, beyond simply PD-L1 co-expression. Malignant lesion [^18^F]FDG SUV-based parameters alone are therefore unlikely to be sufficient in predicting PD-L1 positivity and, as such, anti-PD-1/PD-L1 therapeutic response. However, they have the potential to play an additive role, for example as part of a nomogram taking into account several other multi-modal predictive variables. Several studies have reported the potential utility of SUV-based parameters in predicting PD-L1 expression in the primary tumour and/or anti-PD-1/PD-L1 therapy response, primarily in advanced disease [16–24]. A large study (*n* = 374) of non-selected all-stage NSCLC reported SUV_max_ > 12.5 was predictive of PD-L1 expression, with a sensitivity of 65.4% and specificity of 86.7% [23]. Across the literature, SUV-based parameters, particularly SUV_max_, appear to be most consistently associated with PD-L1, despite the heterogeneity of patient and histological characteristics within and between studies. This is unsurprising given that several preclinical studies have demonstrated that PD-L1 itself promotes expression of GLUT1 and HK2, and thus glycolysis [14, 30].

There are a small number of studies reporting an association of [^18^F]FDG-PET/CT parameters in early-stage lung cancer with PD-L1, but only in the primary tumour. For example, Kaira et al demonstrated the association of high PD-L1 with [^18^F]FDG uptake using SUV_max_ in 315 patients with lung adenocarcinoma [31]. However, the study appeared to use multiple assays and a non-clinically validated scoring mechanism, defining PD-L1 high as membranous staining ≥ 6%. Another study of 548 patients with resected NSCLC demonstrated that pre-operative SUV_max_ was significantly higher in those with a PD-L1 expression ≥ 5% using the SP142 assay [32]. Whilst several studies have shown comparability of most PD-L1 assays (22C3, 28-8, SP263), the SP142 assay does not appear to be comparable, with fewer stained tumour cells [33]. Additionally, the approved cut-off for positivity of PD-L1 expression in NSCLC is either ≥ 50% tumour cells or ≥ 10% immune cells. With a lower, ≥ 5%, cut-off used in this study, it is unclear whether the association of higher SUV_max_ with PD-L1 positivity described has clinical utility.

[^18^F]FDG-PET/CT measures of tumour burden have generally been investigated in terms of immune checkpoint inhibitor response than with PD-L1 expression. Similar to our findings, a study of 32 patients with advanced NSCLC described higher whole-body MTV and TLG in those with positive PD-L1 expression, but not statistically different in the small sample [19]. This is unsurprising as nodal staging in particular focuses on number and location of involved lymph nodes, rather than size, so it is possible to get a wide spectrum of nodal metabolic volumes, i.e. tumour burden, even in early-stage disease. Smaller disease volumes, associated with early-stage disease, are also more prone to partial volume effects and an underestimation of activity concentrations in the analysed image reconstructions [34].

The 1% threshold for PD-L1 positivity in NSCLC is of particular importance considering that several clinical trials have demonstrated that it is associated with clinical benefit to anti-PD-1/PD-L1 therapies in the perioperative, neoadjuvant and adjuvant settings [5, 10, 28]. Our study was conducted in a retrospective cohort preceding routine anti-PD-1/PD-L1 therapy use in early-stage NSCLC, and as such, it was not possible to determine whether these metabolic parameters could predict response and survival associated with anti-PD-1/PD-L1 treatment. This has, however, been demonstrated in advanced NSCLC in several studies [17–19, 35–39]. For example, Takada et al, in 89 patients with advanced NSCLC receiving anti-PD-1 therapy, found that pre-treatment SUV_max_ ≥ 11.16 was significantly associated with higher response rate (41.3%) compared to those with an SUV_max_ < 11.16 (11.6%) [17]. However, the studies are of small samples and there are conflicting results which add additional complexity to understanding the prognostic role these imaging biomarkers could play, in what is an increasingly diverse multi-modality treatment landscape.

Alternative approaches, such as radiomics or deep learning, provide further opportunity. For example, a heterogenous (all-stage) study of 334 patients with NSCLC demonstrated improved prediction of PD-L1 expression using a radiomics model derived from two optimal features extracted from [^18^F]FDG PET and CT components [40]. It may also be of interest to combine those [^18^F]FDG SUV-based parameters associated with PD-L1 expression with other markers of the immune microenvironment, for prediction of anti-PD-1/PD-L1 therapy response. These could include, within a predictive nomogram, SUV-based bone marrow-to-spleen ratio, a surrogate marker of haematopoiesis and immune function, histopathological assessment of tumour infiltrating lymphocytes, tumour mutation burden, a genomic marker of immunogenicity and even clinical factors, such as, smoking status [41–43].

Our study has some limitations. Firstly, this is a single-centre retrospective study, mitigated by the fact that our tertiary centre covers referrals from a wide geographical location across South-East England. Secondly, the SUV was derived from a fixed threshold volume of interest and is therefore potentially affected by the partial volume effect in smaller lesions. Despite the large cohort, the number of PET measurable lymph nodes was limited (*n* = 91) and, as such, it would be important to validate the study’s findings in a larger cohort; this is especially important as these small lesions are more adversely affected by partial volume effect [34]. [^18^F]FDG-PET/CT was performed up to 3 months prior to surgery in this study; there is potential for differences in tumour microenvironment PD-L1 expression at the time of imaging and at tissue retrieval. Of note, we only investigated the association of [^18^F]FDG-PET/CT metabolic parameters with PD-L1 expression as TPS, i.e. tumour cell expression, and using one assay. The SUV measurements of primary tumour and nodal metastases are in fact indicative of [^18^F]FDG uptake within the wider tumour microenvironment, which includes other immune cells, such as tumour infiltrating lymphocytes, that use glycolytic metabolism. As such, it would be useful for further studies to investigate the association of said metabolic parameters with total PD-L1 expression (i.e. tumour and immune cells) and across PD-L1 assays.

## Conclusions

With the ever-increasing role of immune checkpoint inhibitors in the perioperative setting of NSCLC, it is important to understand the heterogeneity and role of PD-L1 expression in early-stage disease, and [^18^F]FDG-PET/CT may play an important role. This study demonstrated an association of standard [^18^F]FDG-PET/CT metabolic parameters with PD-L1 expression in both primary tumour and lymph node metastasis of resectable NSCLC. However, the sensitivity and specificity of these measurements for predicting PD-L1 positivity using the 1% threshold were poor. Future prospective studies are warranted to definitively determine the association, predictive role and, as such, clinical utility of these [^18^F]FDG-PET/CT-based biomarkers for PD-L1 expression and/or PD-L1-directed therapy. Due to the complexity of the tumour microenvironment, it is unlikely that non-invasive [^18^F]FDG-PET/CT metabolic parameters will replace immunohistochemical techniques, or other direct PD-L1 measurement methods alone, but they may supplement them, for example as part of a predictive marker nomogram alongside patient and biological characteristics.

### Supplementary Information

Below is the link to the electronic supplementary material.

Supplementary file1 (PDF 754 KB)

## Abbreviations

[^18^F]FDG: Fluorine 18 fluorodeoxyglucose
CT: Computed tomography
GLUT1: Glucose transporter 1
HIF-1α: Hypoxia-inducible factor 1-alpha
HISUV: SUV-based heterogeneity index
HK2: Hexokinase II
MTV: Metabolic tumour volume
NSCLC (NOS): Non-small cell lung cancer (not otherwise specified)
PD-1: Programmed cell death protein 1
PD-L1: Programmed death-ligand 1
PET: Positron emission tomography
SUL: SUV adjusted for lean body mass
SUV: Standardised uptake value
TLG: Total lesion glycolysis
TPS: Tumour proportion score
VOI: Volume of interest

## Declarations

### Guarantor

The scientific guarantor of this publication is Professor Gary Cook (GJRC).

### Statistics and biometry

One of the authors has significant statistical expertise. No complex statistical methods were necessary for this paper.

### Study subjects or cohort overlap

No study subjects have been previously reported to the best of the authors’ knowledge.

### Methodology

• This is a retrospective observational study performed at one institution involving individual subjects from several referring centres.
