# Supplementary material for: Predicting programmed death-ligand 1 (PD-L1) expression with fluorine-18 fluorodeoxyglucose ([18F]FDG) positron emission tomography/computed tomography (PET/CT) metabolic parameters in resectable non-small cell lung cancer
Source: Eur Radiol. 2024 Feb 22;34(9):5889–902. doi: 10.1007/s00330-024-10651-5 (PMC11364571; doi:10.1007/s00330-024-10651-5)
Supplement: Supplementary file 1 — Supplementary file1 (PDF 754 KB) [file 330_2024_10651_MOESM1_ESM.pdf]

Predicting programmed death-ligand 1 (PD-L1) expression with fluorine-18 fluorodeoxyglucose ([18F]FDG) positron emission tomography/computed tomography (PET/CT) metabolic parameters in resectable non-small cell lung cancer.

ELECTRONIC SUPPLEMENTARY MATERIAL

Supplementary Table 1. Clinical characteristics of all included patients with non-small cell lung cancer by lymph node metastasis PD-L1 tumour proportion score. *P* values represent between TPS group comparisons.

**Clinical characteristic**	**All**	**Lymph node metastasis PD-L1 <1%**	**Lymph node metastasis PD-L1 1-49%**	**Lymph node metastasis PD-L1 ≥50%**	***p value***
**Number (% of total)**	**210 (100)**	133	60	17	
**Age at surgery**					0.99
mean (yrs)	**69**	69	68	69	
median (yrs)	**70**	71	69	68	
range (yrs)	**40-89**	45-89	40-86	54-83	
**Sex, n (%)**					0.38
Female	**90 (43)**	55 (41)	25 (42)	10 (59)	
Male	**120 (57)**	78 (59)	35 (58)	7 (41)	
**Smoking history, n (%)**					0.46
Current smoker	**186 (89)**	121 (91)	49 (81)	16 (94)	
Ex smoker	**8 (4)**	3 (2)	4 (7)	1 (6)	
Never smoker	**10 (5)**	6 (5)	4 (7)	0 (0)	
Unknown	**6 (3)**	3 (2)	3 (5)	0 (0)	
**Histopathology, n (%)**					0.28
Non-squamous*	**143 (68)**	90 (68)	44 (73)	9 (53)	
Squamous cell carcinoma	**67 (32)**	43 (32)	16 (27)	8 (47)	
**Primary tumour PD-L1 score, n (%)**					**<0.001**
<1	**111 (53)**	103 (77)	7 (12)	1 (6)	
1-49	**74 (35)**	25 (19)	44 (73)	5 (29)	
≥50	**25 (12)**	5 (4)	9 (15)	11 (65)	

**Primary tumour location, n (%)**					0.24
Left	**88 (42)**	50 (38)	29 (48)	9 (53)	
Right	**122 (58)**	83 (62)	31 (52)	8 (47)	
**Lymphovascular invasion, n (%)**					0.33
Yes	**108 (51)**	64 (48)	36 (60)	8 (47)	
No	**100 (48)**	67 (50)	24 (40)	9 (53)	
unknown	**2 (1)**	2 (2)	0 (0)	0 (0)	
**Primary tumour max diameter (mm)**					0.62
Median	**40**	38	43	40	
Range	**5 - 150**	10-150	8-98	5-90	
**Pathological T stage, n (%)**					0.86
1(a-c)	**38 (18)**	26 (19)	8 (13)	4 (24)	
2(a-b)	**101 (48)**	62 (47)	32 (53)	7 (41)	
3	**50 (24)**	32 (24)	13 (22)	5 (29)	
4	**21 (10)**	13 (10)	7 (12)	1 (6)	
**Pathological N stage, n (%)**					0.71
1	**118 (56)**	73 (55)	35 (58)	11 (65)	
2	**92 (44)**	60 (45)	25 (42)	6 (35)	
**Pathological M stage, n (%)**					0.76
M0	**208 (99)**	132 (99)	59 (98)	17 (100)	
M1a	**2 (1)**	1 (1)	1 (2)	0 (0)	
**Pathological stage, n (%)**					0.61
IIB	**81 (39)**	47 (35)	27 (45)	7 (41)	
IIIA	**96 (46)**	67 (50)	21 (35)	8 (47)	
IIIB	**31 (15)**	18 (14)	11 (18)	2 (12)	
IVA	**2 (1)**	1 (1)	1 (2)	0 (0)	

**Non-squamous includes:* adenocarcinoma, adenosquamous carcinoma, large cell carcinoma, NSCLC-not otherwise specified.

Supplementary Table 2. Clinical characteristics of all included patients with non-small cell lung cancer by primary tumour PD-L1 TPS above or below the 1% threshold for positive expression. *P* values represent between TPS group comparisons.

**Clinical characteristic**	**All**	**Primary tumour PD-L1 <1%**	**Primary tumour PD-L1 ≥1%**	***p value***
**Number (% of total)**	**210 (100)**	111	99	
**Age at surgery**				0.67
mean (yrs)	**69**	69	69	
median (yrs)	**70**	70	70	
range (yrs)	**40-89**	45-89	40-86	
**Sex, n (%)**				0.66
Female	**90 (43)**	46 (41)	44 (44)	
Male	**120 (57)**	65 (59)	55 (56)	
**Smoking history, n (%)**				0.11
Current smoker	**186 (89)**	98 (88)	88 (89)	
Ex smoker	**8 (4)**	4 (4)	4 (4)	
Never smoker	**10 (5)**	8 (7)	2 (2)	
Unknown	**6 (3)**	1 (1)	5 (5)	
**Histopathology, n (%)**				0.67
Non-squamous*	**143 (68)**	77 (69)	66 (67)	
Squamous cell carcinoma	**67 (32)**	34 (31)	33 (33)	
**Lymph node PD-L1 score, n (%)**				**<0.001**
<1	**133 (63)**	103 (93)	30 (30)	
1-49	**60 (29)**	7 (6)	54 (55)	
≥50	**17 (8)**	1 (1)	16 (16)	
**Primary tumour location, n (%)**				0.67
Left	**88 (42)**	45 (41)	43 (43)	
Right	**121 (58)**	66 (59)	56 (57)	
**Lymphovascular invasion, n (%)**				0.66
Yes	**108 (51)**	55 (49)	53 (54)	
No	**100 (48)**	54 (49)	46 (46)	
unknown	**2 (1)**	2 (2)	0 (0)	
**Primary tumour max diameter (mm)**				0.57
Median	**40**	38	40	
Range	**5 - 150**	11 - 150	5 - 98	

**Pathological T stage, n (%)**				0.68
1(a-c)	**38 (18)**	22 (20)	16 (16)	
2(a-b)	**101 (48)**	49 (44)	52 (53)	
3	**50 (24)**	28 (25)	22 (22)	
4	**21 (10)**	12 (11)	9 (9)	
**Pathological N stage, n (%)**				0.70
1	**118 (56)**	61 (55)	57 (58)	
2	**92 (44)**	50 (45)	42 (42)	
**Pathological M stage, n (%)**				0.94
M0	**208 (99)**	110 (99)	98 (99)	
M1a	**2 (1)**	1 (1)	1 (1)	
**Pathological stage, n (%)**				0.85
IIB	**81 (39)**	40 (36)	41 (41)	
IIIA	**96 (46)**	52 (47)	44 (45)	
IIIB	**31 (15)**	18 (16)	13 (13)	
IVA	**2 (1)**	1 (1)	1 (1)	

**Non-squamous includes:* adenocarcinoma, adenosquamous carcinoma, large cell carcinoma, NSCLC-not otherwise specified.

Supplementary Table 3. Clinical characteristics of all included patients with non-small cell lung cancer by lymph node metastasis PD-L1 TPS above or below the 1% threshold for positive expression. *P* values represent between TPS group comparisons.

**Clinical characteristic**	**All**	**Lymph node metastasis PD-L1 <1%**	**Lymph node metastasis PD-L1 ≥1%**	***p value***
**Number (% of total)**	**210 (100)**	133	77	
**Age at surgery**				0.87
mean (yrs)	**69**	69	69	
median (yrs)	**70**	71	69	
range (yrs)	**40-89**	45-89	40-86	
**Sex, n (%)**				0.56
Female	**90 (43)**	55 (41)	35 (45)	
Male	**120 (57)**	78 (59)	42 (55)	
**Smoking history, n (%)**				0.38
Current smoker	**186 (89)**	121 (91)	65 (85)	
Ex smoker	**8 (4)**	3 (2)	5 (6)	
Never smoker	**10 (5)**	6 (5)	4 (5)	
Unknown	**6 (3)**	3 (2)	3 (4)	
**Histopathology, n (%)**				0.86
Non-squamous*	**143 (68)**	90 (68)	53 (69)	
Squamous cell carcinoma	**67 (32)**	43 (32)	24 (31)	
**Primary tumour PD-L1 score, n (%)**				**<0.001**
<1	**111 (53)**	103 (77)	8 (10)	
1-49	**74 (35)**	25 (19)	49 (64)	
≥50	**25 (12)**	5 (4)	20 (26)	
**Primary tumour location, n (%)**				0.10
Left	**88 (42)**	50 (38)	38 (49)	
Right	**121 (58)**	83 (62)	39 (51)	
**Lymphovascular invasion, n (%)**				0.29
Yes	**108 (51)**	64 (48)	44 (57)	
No	**100 (48)**	67 (50)	33 (43)	
unknown	**2 (1)**	2 (2)	0 (0)	
**Primary tumour max diameter (mm)**				0.33
Median	**40**	38	42	

Range	**5 - 150**	10 - 150	5 - 98	
**Pathological T stage, n (%)**				0.89
1(a-c)	**38 (18)**	26 (19)	12 (16)	
2(a-b)	**101 (48)**	62 (47)	39 (51)	
3	**50 (24)**	32 (24)	18 (23)	
4	**21 (10)**	13 (10)	8 (10)	
**Pathological N stage, n (%)**				0.49
1	**118 (56)**	73 (55)	46 (60)	
2	**92 (44)**	60 (45)	31 (40)	
**Pathological M stage, n (%)**				0.69
M0	**208 (99)**	132 (99)	76 (99)	
M1a	**2 (1)**	1 (1)	1 (1)	
**Pathological stage, n (%)**				0.36
IIB	**81 (39)**	47 (35)	34 (44)	
IIIA	**96 (46)**	67 (50)	29 (38)	
IIIB	**31 (15)**	18 (14)	13 (17)	
IVA	**2 (1)**	1 (1)	1 (1)	

**Non-squamous includes:* adenocarcinoma, adenosquamous carcinoma, large cell carcinoma, NSCLC-not otherwise specified.

Supplementary Table 4. Clinical characteristics of all included patients with non-small cell lung cancer by primary tumour PD-L1 TPS above or below 50%. *P* values represent between TPS group comparisons.

**Clinical characteristic**	**All**	**Primary tumour PD-L1 <50%**	**Primary tumour PD-L1 ≥50%**	***p value***
**Number (% of total)**	**210 (100)**	185	25	
**Age at surgery**				0.57
mean (yrs)	**69**	69	70	
median (yrs)	**70**	70	72	
range (yrs)	**40 - 89**	40 - 89	46 – 86	
**Sex, n (%)**				0.58
Female	**90 (43)**	78 (42)	12 (48)	
Male	**120 (57)**	107 (58)	13 (52)	
**Smoking history, n (%)**				0.68
Current smoker	**186 (89)**	163 (88)	23 (92)	
Ex smoker	**8 (4)**	7 (4)	1 (4)	
Never smoker	**10 (5)**	10 (5)	0 (0)	
Unknown	**6 (3)**	5 (3)	1 (4)	
**Histopathology, n (%)**				0.37
Non-squamous*	**143 (68)**	124 (67)	19 (76)	
Squamous cell carcinoma	**67 (32)**	61 (33)	6 (24)	
**Primary tumour PD-L1 score, n (%)**				**<0.001**
<1	**133 (63)**	128 (70)	5 (20)	
1-49	**60 (29)**	51 (28)	9 (36)	
≥50	**17 (8)**	6 (3)	11 (44)	
**Primary tumour location, n (%)**				0.51
Left	**88 (42)**	76 (41)	12 (48)	
Right	**121 (58)**	109 (59)	13 (52)	
**Lymphovascular invasion, n (%)**				0.38
Yes	**108 (51)**	92 (50)	16 (64)	
No	**100 (48)**	91 (49)	9 (36)	
unknown	**2 (1)**	2 (1)	0 (0)	
**Primary tumour max diameter (mm)**				0.57
Median	**40**	40	35	
Range	**5 - 150**	5 - 150	10 - 90	

**Pathological T stage, n (%)**				0.17
1(a-c)	**38 (18)**	34 (18)	4 (16)	
2(a-b)	**101 (48)**	84 (45)	17 (68)	
3	**50 (24)**	47 (25)	3 (12)	
4	**21 (10)**	20 (11)	1 (4)	
**Pathological N stage, n (%)**				0.09
1	**118 (56)**	100 (54)	18 (72)	
2	**92 (44)**	85 (46)	7 (28)	
**Pathological M stage, n (%)**				0.60
M0	**208 (99)**	183 (99)	25 (100)	
M1a	**2 (1)**	2 (1)	0 (0)	
**Pathological stage, n (%)**				**0.04**
IIB	**81 (39)**	65 (35)	16 (64)	
IIIA	**96 (46)**	88 (48)	8 (32)	
IIIB	**31 (15)**	30 (16)	1 (4)	
IVA	**2 (1)**	2 (1)	0 (0)	

**Non-squamous includes:* adenocarcinoma, adenosquamous carcinoma, large cell carcinoma, NSCLC-not otherwise specified.

Supplementary Table 5. Clinical characteristics of all included patients with non-small cell lung cancer by lymph node metastasis PD-L1 TPS above or below 50%. *P* values represent between TPS group comparisons.

**Clinical characteristic**	**All**	**Lymph node metastasis PD-L1 <50%**	**Lymph node metastasis PD-L1 ≥50%**	***p value***
**Number (% of total)**	**210 (100)**	193	17	
**Age at surgery**				0.96
mean (yrs)	**69**	69	69	
median (yrs)	**70**	70	68	
range (yrs)	**40 - 89**	40 - 89	54 - 83	
**Sex, n (%)**				0.17
Female	**90 (43)**	80 (41)	10 (59)	
Male	**120 (57)**	113 (59)	7 (41)	
**Smoking history, n (%)**				0.64
Current smoker	**186 (89)**	170 (88)	16 (94)	
Ex smoker	**8 (4)**	7 (4)	1 (6)	
Never smoker	**10 (5)**	10 (5)	0 (0)	
Unknown	**6 (3)**	6 (3)	0 (0)	
**Histopathology, n (%)**				0.16
Non-squamous*	**143 (68)**	134 (69)	9 (53)	
Squamous cell carcinoma	**67 (32)**	59 (31)	8 (47)	
**Primary tumour PD-L1 score, n (%)**				**<0.001**
<1	**111 (53)**	110 (57)	1 (6)	
1-49	**74 (35)**	69 (36)	5 (29)	
≥50	**25 (12)**	14 (7)	11 (65)	
**Primary tumour location, n (%)**				0.34
Left	**88 (42)**	79 (41)	9 (53)	
Right	**121 (58)**	114 (59)	8 (47)	
**Lymphovascular invasion, n (%)**				0.84
Yes	**108 (51)**	100 (52)	8 (47)	
No	**100 (48)**	91 (47)	9 (53)	
unknown	**2 (1)**	2 (1)	0 (0)	
**Primary tumour max diameter (mm)**				0.68

Median	**40**	39	40	
Range	**5 - 150**	8 - 150	5 - 90	
**Pathological T stage, n (%)**				0.79
1(a-c)	**38 (18)**	34 (18)	4 (24)	
2(a-b)	**101 (48)**	94 (49)	7 (41)	
3	**50 (24)**	45 (23)	5 (29)	
4	**21 (10)**	20 (10)	1 (6)	
**Pathological N stage, n (%)**				0.49
1	**118 (56)**	108 (56)	11 (65)	
2	**92 (44)**	85 (44)	6 (35)	
**Pathological M stage, n (%)**				0.67
M0	**208 (99)**	191 (99)	17 (100)	
M1a	**2 (1)**	2 (1)	0 (0)	
**Pathological stage, n (%)**				0.95
IIB	**81 (39)**	74 (38)	7 (41)	
IIIA	**96 (46)**	88 (46)	8 (47)	
IIIB	**31 (15)**	29 (15)	2 (12)	
IVA	**2 (1)**	2 (1)	0 (0)	

**Non-squamous includes:* adenocarcinoma, adenosquamous carcinoma, large cell carcinoma, NSCLC-not otherwise specified.

Supplementary Table 6. Multivariate analysis of the relationship of PD-L1 expression (< or ≥1%) and selected factors by logistic regression. Estimates of the odds ratios are shown with their 95% confidence interval (CI) and associated *p* values.

	Factor	Odds ratio	95% confidence interval	*P* value
**Primary tumour**	Age	1.01	0.98 – 1.04	0.72
	Sex	1.25	0.69 – 2.25	0.47
	Smoking	0.31	0.05 – 1.34	0.16
	Location	0.92	0.51 – 1.66	0.78
	Histology	1.12	0.57 – 2.23	0.74
	pT1 status	1.02	0.48 – 2.20	0.95
	pN1 status	0.81	0.45 – 1.45	0.48
	SUV_{max}	0.94	0.69 – 1.27	0.70
	SUV_{mean}	1.21	0.75 – 2.00	0.43
**Lymph node**	Age	1.09	1.03 – 1.16	**0.005**
	Sex	1.88	0.68 – 5.38	0.23
	Smoking	6.90	0.41 – 200.70	0.19
	Location	1.37	0.50 – 3.78	0.53
	Histology	1.52	0.52 – 4.62	0.45
	pT1 status	0.75	0.19 – 3.02	0.68
	pN1 status	1.03	0.37 – 2.90	0.95
	SUV_{max}	0.76	0.49 – 1.09	0.16
	SUV_{mean}	2.17	1.14 – 4.95	**0.03**

Supplementary Table 7. Mean metabolic parameter scores and their standard error (SE), for both primary lung tumour and involved lymph nodes, by PD-L1 TPS above or below the 50% threshold for positive expression. The *n* varies between groups and individual parameters, for example, peak is only measurable in lesions with a minimum 1 cm^3 volume. Mann-Whitney *p* values presented for each parameter in primary lung tumour or lymph node metastasis (significant in bold).

PET metabolic parameter	PD-L1 TPS <50%	PD-L1 TPS ≥50%	*Mann-Whitney p value*
**SUV_{max}**			
Primary tumour, *n*	185	25	
mean (SE)	12.52 (0.47)	15.19 (1.32)	**<0.05**
Lymph node, *n*	81	10	
mean (SE)	6.61 (0.42)	11.50 (2.64)	0.07
**SUV_{mean}**			
Primary tumour, *n*	182	25	
mean (SE)	7.65 (0.29)	9.23 (0.82)	0.05
Lymph node, *n*	79	10	
mean (SE)	4.21 (0.24)	7.48 (1.71)	**<0.05**
**SUV_{peak}**			
Primary tumour, *n*	165	23	
mean (SE)	11.38 (0.44)	13.44 (1.30)	0.14
Lymph node, *n*	36	4	
mean (SE)	6.55 (0.60)	18.36 (2.76)	**<0.001**
**SUL_{peak}**			
Primary tumour, *n*	162	23	
mean (SE)	8.31 (0.33)	9.65 (0.90)	0.16
Lymph node, *n*	34	4	
mean (SE)	4.95 (0.50)	13.31 (2.45)	**0.001**
**MTV**			
Primary tumour, *n*	182	25	
mean (SE)	25.05 (2.72)	20.74 (5.28)	0.60
Lymph node, *n*	79	10	
mean (SE)	3.32 (0.38)	11.79 (7.19)	0.42
**TLG**			
Primary tumour, *n*	182	25	
mean (SE)	217.20 (24.85)	221.00 (59.28)	0.80
Lymph node, *n*	79	10	
mean (SE)	15.93 (2.47)	117.40 (66.42)	0.28
**HISUV**			
Primary tumour, *n*	182	25	
mean (SE)	1.66 (0.01)	1.66 (0.02)	0.62
Lymph node, *n*	79	10	
mean (SE)	1.54 (0.03)	1.53 (0.07)	>0.99

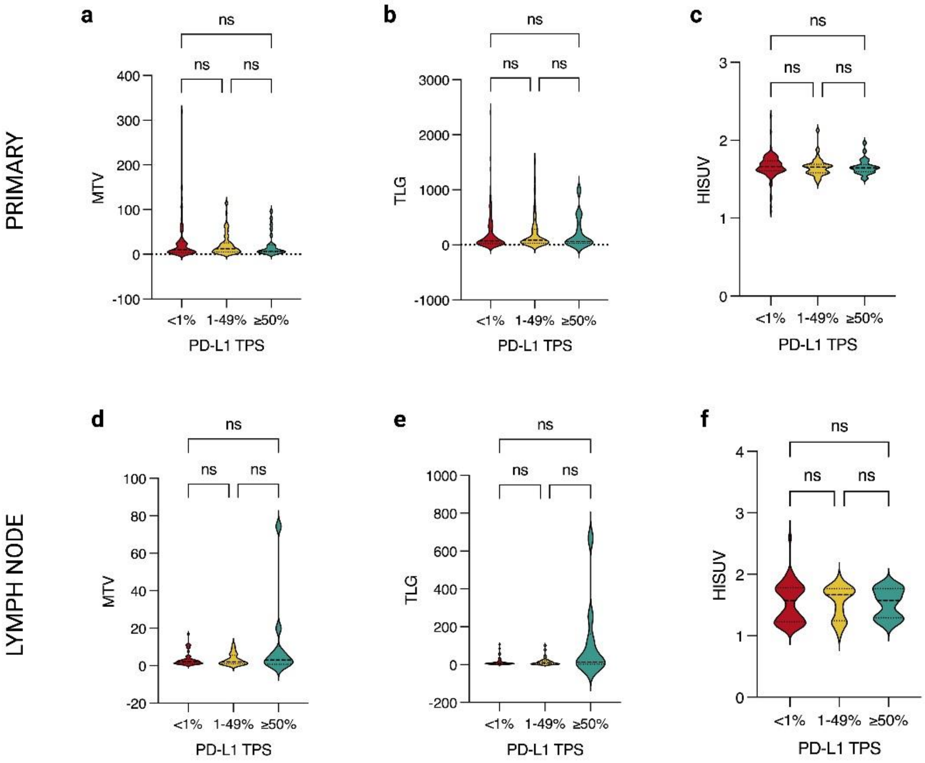

Supplementary Figure 1. Violin plots displaying primary lung tumour (a-c) and malignant lymph node (d-f) metabolic parameter median and lower/upper quartiles (dashed lines) of PD-L1 TPS groups of <1%, 1-49% and ≥50%. Metabolic parameters of (**a**) primary MTV ($p = 0.80$, H test = 0.44), (**b**) primary TLG ($p = 0.82$, H test = 0.39), (**c**) primary HISUV ($p = 0.30$, H test = 2.39), (**d**) lymph node MTV ($p = 0.71$, H test = 0.68), (**e**) lymph node TLG ($p = 0.47$, H test = 1.52), and (**f**) lymph node HISUV ($p = 0.97$, H test = 0.06). Lines above the plots demonstrate the Dunn's multiple comparison tests between two individual TPS groups, where ns is non-significant ($p > 0.05$) and ***** represents the level of significance (* is $p < 0.05$; ** is $p < 0.01$, and *** is $p < 0.001$). *Individual graphs created in GraphPad Prism, figure created with BioRender.com.*

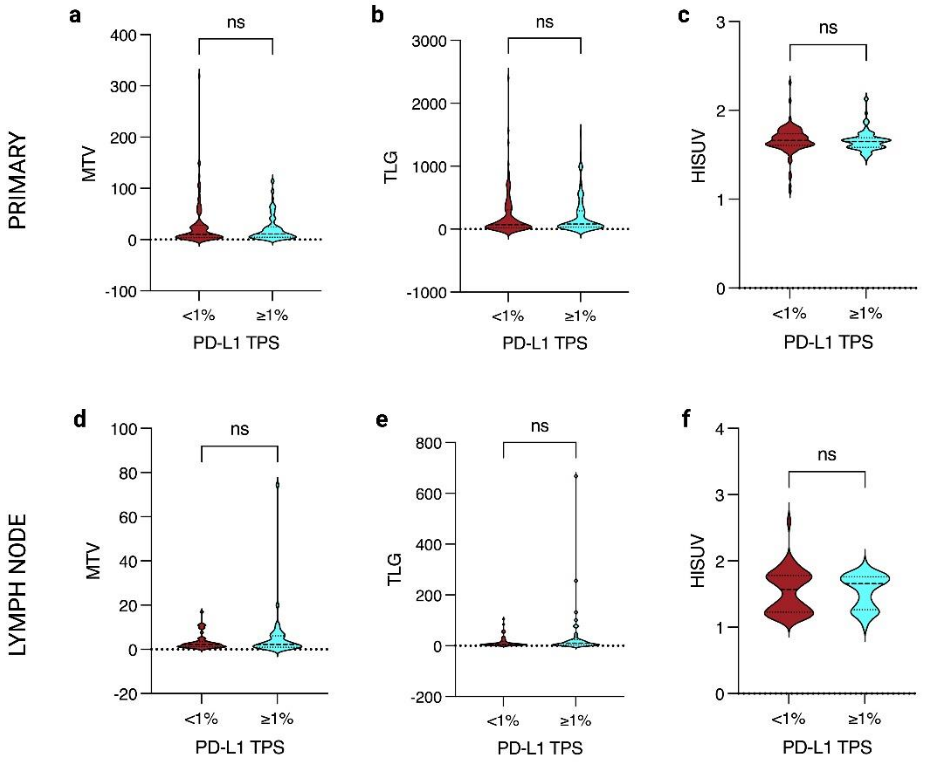

Supplementary Figure 2. Violin plots displaying primary lung tumour (a-c) and malignant lymph node (d-f) metabolic parameter median and lower/upper quartiles (dashed lines) of PD-L1 TPS groups of <1% and ≥1%. Metabolic parameters of (**a**) primary MTV (p = 0.88), (**b**) primary TLG (p = 0.54), (**c**) primary HISUV (p = 0.12), (**d**) lymph node MTV (p = 0.81), (**e**) lymph node TLG (p = 0.34), and (**f**) lymph node HISUV (p = 0.83). Lines above the plots demonstrate the Mann-Whitney U test p values between the two TPS groups, where ns is non-significant ($p > 0.05$) and ***** represents the level of significance (* is $p < 0.05$; ** is $p < 0.01$, and *** is $p < 0.001$). *Individual graphs created in GraphPad Prism, figure created with BioRender.com.*

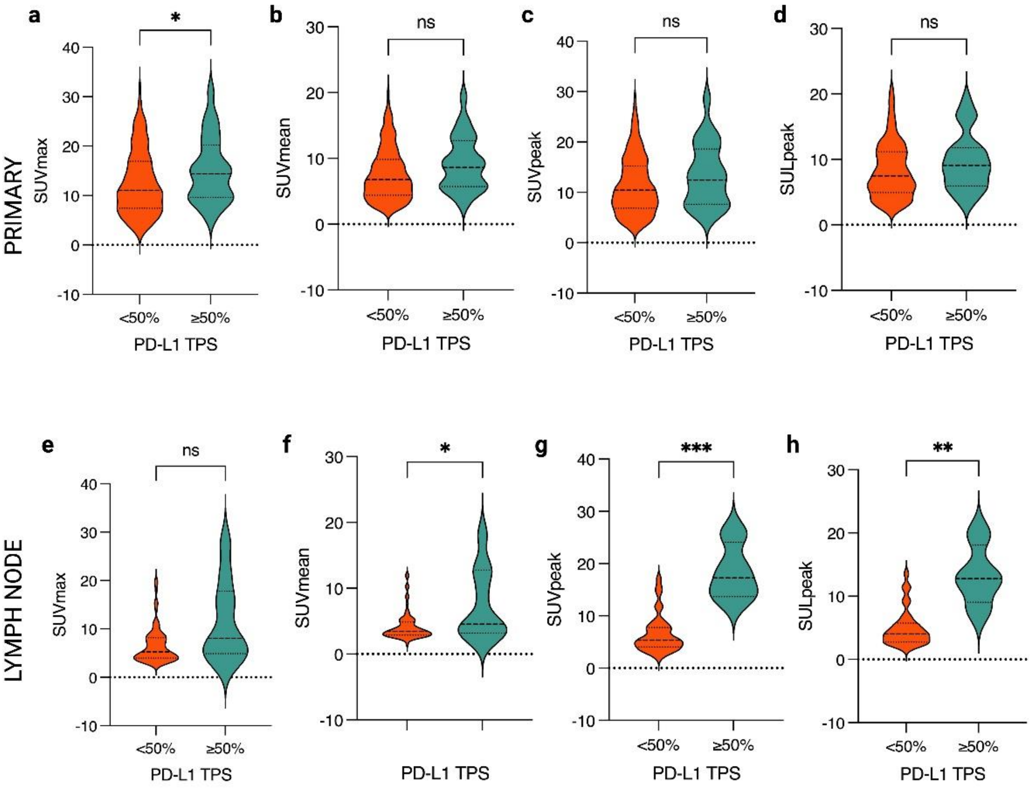

Supplementary Figure 3. Violin plots displaying primary lung tumour (a-d) and malignant lymph node (e-h) metabolic parameter median and lower/upper quartiles (dashed lines) of PD-L1 TPS groups of <50% and ≥50%. Metabolic parameters of (**a**) primary SUV$_{max}$ (p <0.05), (**b**) primary SUV$_{mean}$ (p = 0.05), (**c**) primary SUV$_{peak}$ (p = 0.14), (**d**) primary SUL$_{peak}$ (p = 0.16), (**e**) lymph node SUV$_{max}$ (p = 0.07), (**f**) lymph node SUV$_{mean}$ (p <0.05), (**g**) lymph node SUV$_{peak}$ (p <0.05), and (**h**) lymph node SUL$_{peak}$ (p <0.05). Lines above the plots demonstrate the Mann-Whitney U test p values between the two TPS groups, where ns is non-significant (p > 0.05) and ***** represents the level of significance (* is p < 0.05). *Individual graphs created in GraphPad Prism, figure created with BioRender.com.*

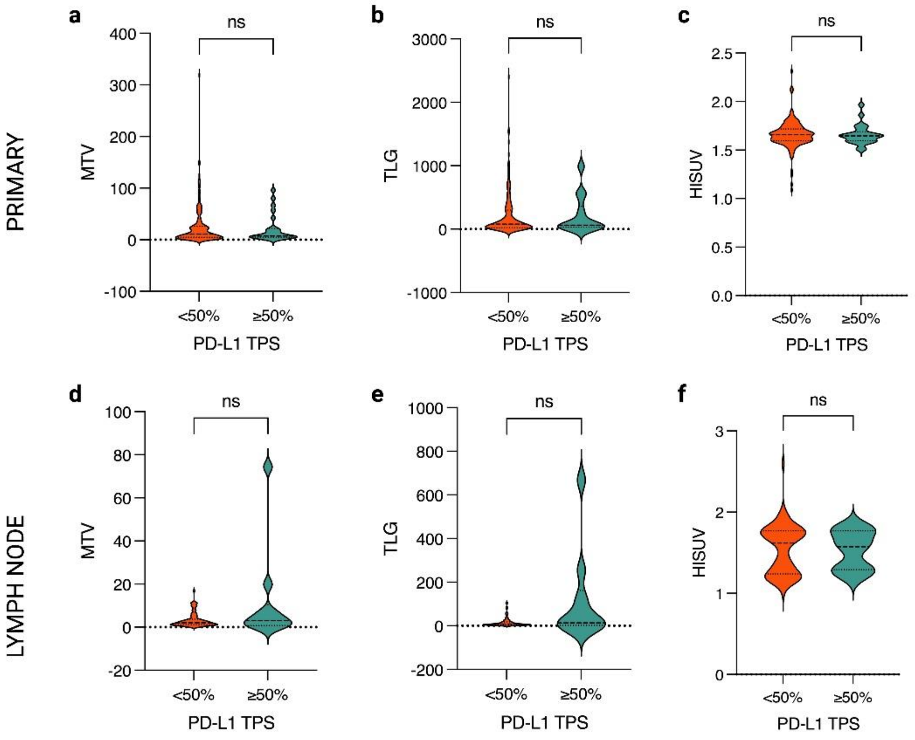

Supplementary Figure 4. Violin plots displaying primary tumour (a-c) and malignant lymph node (d-f) metabolic parameter median and lower/upper quartiles (dashed lines) of PD-L1 TPS groups of <50% and ≥50%. Metabolic parameters of (**a**) primary MTV (p = 0.60), (**b**) primary TLG (p = 0.80), (**c**) primary HISUV (p = 0.62), (**d**) lymph node MTV (p = 0.42), (**e**) lymph node TLG (p = 0.28), and (**f**) lymph node HISUV (p >0.99). Lines above the plots demonstrate the Mann-Whitney U test p values between the two TPS groups, where ns is non-significant (p > 0.05) and ***** represents the level of significance (* is p < 0.05; ** is p < 0.01, and *** is p < 0.001). *Individual graphs created in GraphPad Prism, figure created with BioRender.com.*

SUPPLEMENTARY FIGURE AND TABLE LEGENDS

Supplementary Table 1. Clinical characteristics of all included patients with non-small cell lung cancer by lymph node metastasis PD-L1 tumour proportion score. *P* values represent between TPS group comparisons.

Supplementary Table 2. Clinical characteristics of all included patients with non-small cell lung cancer by primary tumour PD-L1 TPS above or below the 1% threshold for positive expression. *P* values represent between TPS group comparisons.

Supplementary Table 3. Clinical characteristics of all included patients with non-small cell lung cancer by lymph node metastasis PD-L1 TPS above or below the 1% threshold for positive expression. *P* values represent between TPS group comparisons.

Supplementary Table 4. Clinical characteristics of all included patients with non-small cell lung cancer by primary tumour PD-L1 TPS above or below 50%. *P* values represent between TPS group comparisons.

Supplementary Table 5. Clinical characteristics of all included patients with non-small cell lung cancer by lymph node metastasis PD-L1 TPS above or below 50%. *P* values represent between TPS group comparisons.

Supplementary Table 6. Multivariate analysis of the relationship of PD-L1 expression (< or ≥1%) and selected factors by logistic regression. Estimates of the odds ratios are shown with their 95% confidence interval (CI) and associated *p* values.

Supplementary Table 7. Mean metabolic parameter scores and their standard error (SE), for both primary lung tumour and involved lymph nodes, by PD-L1 TPS above or

below the 50% threshold for positive expression. The *n* varies between groups and individual parameters, for example, peak is only measurable in lesions with a minimum 1 cm^3 volume. Mann-Whitney *p* values presented for each parameter in primary lung tumour or lymph node metastasis (significant in bold).

Supplementary Figure 1. Violin plots displaying primary lung tumour (a-c) and malignant lymph node (d-f) metabolic parameter median and lower/upper quartiles (dashed lines) of PD-L1 TPS groups of <1%, 1-49% and ≥50%. Metabolic parameters of (**a**) primary MTV ($p = 0.80$, *H* test = 0.44), (**b**) primary TLG ($p = 0.82$, *H* test = 0.39), (**c**) primary HISUV ($p = 0.30$, *H* test = 2.39), (**d**) lymph node MTV ($p = 0.71$, *H* test = 0.68), (**e**) lymph node TLG ($p = 0.47$, *H* test = 1.52), and (**f**) lymph node HISUV ($p = 0.97$, *H* test = 0.06). Lines above the plots demonstrate the Dunn's multiple comparison tests between two individual TPS groups, where ns is non-significant ($p > 0.05$) and ***** represents the level of significance (* is $p < 0.05$; ** is $p < 0.01$, and *** is $p < 0.001$). *Individual graphs created in GraphPad Prism, figure created with BioRender.com.*

Supplementary Figure 2. Violin plots displaying primary lung tumour (a-c) and malignant lymph node (d-f) metabolic parameter median and lower/upper quartiles (dashed lines) of PD-L1 TPS groups of <1% and ≥1%. Metabolic parameters of (**a**) primary MTV ($p = 0.88$), (**b**) primary TLG ($p = 0.54$), (**c**) primary HISUV ($p = 0.12$), (**d**) lymph node MTV ($p = 0.81$), (**e**) lymph node TLG ($p = 0.34$), and (**f**) lymph node HISUV ($p = 0.83$). Lines above the plots demonstrate the Mann-Whitney U test *p* values between the two TPS groups, where ns is non-significant ($p > 0.05$) and ***** represents the level of significance (* is $p < 0.05$; ** is $p < 0.01$, and *** is $p < 0.001$). *Individual graphs created in GraphPad Prism, figure created with BioRender.com.*

Supplementary Figure 3. Violin plots displaying primary lung tumour (a-d) and malignant lymph node (e-h) metabolic parameter median and lower/upper quartiles

(dashed lines) of PD-L1 TPS groups of <50% and ≥50%. Metabolic parameters of (**a**) primary SUV_{max} (*p* <0.05), (**b**) primary SUV_{mean} (*p* = 0.05), (**c**) primary SUV_{peak} (*p* = 0.14), (**d**) primary SUL_{peak} (*p* = 0.16), (**e**) lymph node SUV_{max} (*p* = 0.07), (**f**) lymph node SUV_{mean} (*p* <0.05), (**g**) lymph node SUV_{peak} (*p* <0.05), and (**h**) lymph node SUL_{peak} (*p* <0.05). Lines above the plots demonstrate the Mann-Whitney U test *p* values between the two TPS groups, where ns is non-significant (*p* > 0.05) and ***** represents the level of significance (* is *p* < 0.05). *Individual graphs created in GraphPad Prism, figure created with BioRender.com.*

Supplementary Figure 4. Violin plots displaying primary tumour (a-c) and malignant lymph node (d-f) metabolic parameter median and lower/upper quartiles (dashed lines) of PD-L1 TPS groups of <50% and ≥50%. Metabolic parameters of (**a**) primary MTV (*p* = 0.60), (**b**) primary TLG (*p* = 0.80), (**c**) primary HISUV (*p* = 0.62), (**d**) lymph node MTV (*p* = 0.42), (**e**) lymph node TLG (*p* = 0.28), and (**f**) lymph node HISUV (*p* >0.99). Lines above the plots demonstrate the Mann-Whitney U test *p* values between the two TPS groups, where ns is non-significant (*p* > 0.05) and ***** represents the level of significance (* is *p* < 0.05; ** is *p* < 0.01, and *** is *p* < 0.001). *Individual graphs created in GraphPad Prism, figure created with BioRender.com.*
